# OP52 How Will European Joint Clinical Assessment Impact National Decision-Making?

**DOI:** 10.1017/S0266462324001144

**Published:** 2025-01-07

**Authors:** Elvira Müller, Kurt Neeser

## Abstract

**Introduction:**

In 2025, oncology drugs with new active substances and advanced therapy medicinal products will undergo joint clinical assessment (JCA). The comparative analysis of the clinical evidence as defined in the Regulation (EU) 2021/2282 on health technology assessment (HTAR) will save national/regional submissions of the same evidence. JCA will be available early supporting appraisal and decision-making, which remains within the responsibility of member states (MS).

**Methods:**

Targeted searches on JCA and statements from stakeholders were performed and analyzed. We conducted interviews with current and former national payers, as well as members of HTA agencies, across Germany, France, Italy, and Eastern Europe to explore their perspectives on the anticipated implications of JCA on decision-making processes and reimbursement strategies in Europe. Focus was on reduced/additional effort for authorities and health technology developers (HTDs), required national amendments, and potential discrepancies between JCA outcome and MS benefit evaluations.

**Results:**

Stakeholders appreciate the standardized methodology and guidance on HTA, which, especially in countries without an established HTA system, could enhance patients’ access to new treatments by considering JCA in decision-making. The comprehensive evidence compilation may also save resources in pursuing national/regional submissions. On the other hand, country-based appraisals within the MS could lead to diverse conclusions, and there is uncertainty as to which extent national authorities will adopt JCA and how its integration into decision-making will be handled. Some stakeholders challenge an impact on local patients’ access as reimbursement and pricing processes remain within MS responsibility.

**Conclusions:**

JCA is a long-desired achievement and will set the groundwork for timely access of new treatments in the MS. However, presently there are several uncertainties on how JCA will impact decision-making and whether MS appraisal could lead to contradictory value conclusions for a given treatment. Future adjustments to national/regional procedures and refinement of the JCA framework are expected.

